# Glomerular Diseases Associated with Malignancies: Histopathological Pattern and Association with Circulating Autoantibodies

**DOI:** 10.3390/antib9020018

**Published:** 2020-05-25

**Authors:** Sophia Lionaki, Smaragdi Marinaki, Konstantinos Panagiotellis, Ioanna Tsoumbou, George Liapis, Ioanna Vlahadami, Athanasios Tzioufas, Petros Sfikakis, Ioannis Boletis

**Affiliations:** 1 Nephrology Department & Transplantation Unit, Faculty of Medicine, Laiko Hospital, National & Kapodistrian University of Athens, 11527 Athens, Greece; smaragdimarinaki@yahoo.com (S.M.); panagiotellis.konstantinos@hotmail.gr (K.P.); iotsoumbou@gmail.com (I.T.); laikneph@laiko.gr (I.B.); 2 Pathology Department, Faculty of Medicine, Laiko Hospital, National & Kapodistrian University of Athens, 11527 Athens, Greece; gliapis@gmail.com; 3 Department of Pathophysiology, Faculty of Medicine, Laiko Hospital, National & Kapodistrian University of Athens, 11527 Athens, Greece; ivlah@gmail.com (I.V.); agtz@med.uoa.gr (A.T.); 4 1st Department of Propaedeutic and Internal Medicine and Joined Rheumatology Program, Medical School, Laiko Hospital, National and Kapodistrian University of Athens, 11527 Athens, Greece; psfikakis@med.uoa.gr

**Keywords:** glomerular disease, malignancy, solid tumor, lymphoid disorder, outcome

## Abstract

Aim: Glomerular diseases (GD) associated with malignancies (AM, GDAM) have unique features, which are important to recognize, in the light of the progress made in cancer therapy. We aimed to describe the clinical and histopathological characteristics of patients with GDAM in relation to the presence of circulating autoantibodies, pointing to potential immune pathogenic pathways connecting cancer to GD. Materials and Methods: The included patients were studied retrospectively on the basis of a kidney biopsy proving GD and a related biopsy to establish the diagnosis of AM. We recorded patients’ demographics, serological and laboratory parameters, histopathological findings, and the type of malignancy, GD, and therapy. Results: In total, 41 patients with GDAM, with a mean age of 63.1 (±10.7) years, were studied. In 28 (68.3%) cases, GD was associated with a solid tumor, and in 13 (31.7%) patients with a lymphoid malignancy. The most frequent histopathological pattern was membranous nephropathy (43.9%). Overall, at the time of GD diagnosis, 17% of the patients were positive for antinuclear antibodies (ANA), and 12.2% for antineutrophil cytoplasmic autoantibodies (ANCA), all against myeloperoxidase (MPO). In addition, 93.3% of the patients who had membranous nephropathy were negative for transmembrane glycoprotein M-type phospholipase A_2_ receptor (PLA_2_R) antibody. Sixteen patients (39.0%) presented with acute nephritic syndrome, of whom five (31.25%) developed rapidly progressive glomerulonephritis. In a mean follow-up time of 36.1 (±28.3) months, nine (21.95%) patients ended up with end-stage kidney disease, and eight (19.5%) died. Conclusion: We found that 3.2% of patients who underwent a native kidney biopsy in our institution during the past decade, for any reason, were identified as having some type of GD associated with a malignancy. Serology indicated a significant presence of ANA or MPO-ANCA antibodies in patients with nephritic syndrome and the absence of PLA_2_R antibodies in patients with membranous nephropathy.

## 1. Introduction

Glomerular diseases (GD) represent certain patterns of injury of the glomeruli, which may be associated with an inherited or acquired disorder and manifest with various clinical pictures and grades of severity, from asymptomatic urinary abnormalities to acute kidney insufficiency. Since the glomerulus and its surrounding Bowman’s capsule constitute the basic filtration unit of the kidney, long-standing or aggressive disease causing glomerular changes may result in irreversible kidney damage, chronic renal failure, and end-stage kidney disease. Genetic and environmental factors have been implicated in the pathogenesis of GDs, including infections, medications, and malignancies. GD associated with malignancies (GDAM) represent a rare, secondary form of glomerular lesion and a complication of cancer, which remains a challenge for both nephrologists and oncologists. They are not directly related to the tumor burden, invasion, or metastasis but are assumed to be caused by tumor cell products, such as hormones, growth factors, cytokines, and tumor antigens [1]. Recognition of GDAM is clinically crucial for several reasons. First, if the neoplasm is not known, subsequent detection of an undiagnosed malignancy could be life-saving. Second, if GD is mistaken as idiopathic GD, it can lead to unsuccessful and possibly dangerous therapies. Finally, the pathogenic mechanisms of many glomerular lesions seem to be related to the altered immune responses associated with malignancies and, thus, may facilitate the identification of biomarkers and the investigation of the pathology [2]. The pathogenesis of each type of GDAM is considered to be related to the nature of the respective neoplasm, and therefore, GD associated with solid tumors and lymphoproliferative disorders develop differently. Potential pathogenic mechanisms include in situ formation of immune complexes, with antibodies targeting a tumor antigen localized in the glomeruli, trapping of circulating immune complexes in the glomerular capillaries, and involvement of external factors, such as oncogenic viruses and/or altered immune function [3].

Estimations on the frequency of GDAM are confounded by several factors, including the more aggressive screening for cancer in patients with nephrotic syndrome than in those with nephritic syndrome and the fact that a significant proportion of patients may not present malignancy-related symptoms at the discovery of GD [4]. Besides, certain GDs, such as membranous nephropathy (MN) and pauci-immune glomerulonephritis (PI-GN), occur more often in the elderly, as does cancer, while many of the agents used in the treatment of GD are potentially oncogenic [5]. The management of patients with GDAM is targeted at the primary cause, i.e., the neoplasm, and requires a multidisciplinary approach to monitor both cancer and GD.

The aim of this study was to describe the patterns of renal histopathology in patients with GDAM in relation to serological and clinical features and the type of malignancy. The detection of circulating autoantibodies in serum might also indicate potential immunopathogenic pathways connecting these cancer and GD.

## 2. Materials and Methods

### 2.1. Participants and Inclusion Criteria

We retrospectively reviewed the medical records of all patients who were diagnosed with any type of GDAM in the period 2008–2018 in our hospital. GDAM might have been identified simultaneously with cancer, i.e., during the same admission workup, before, or after the diagnosis of cancer. Participants had to meet the following criteria: (i) histopathological diagnosis of GD in a native kidney biopsy, (ii) GD associated with active and biopsy-proven malignancy (based on a biopsy of the related lesion for solid tumors or a lymph node and/or bone marrow aspiration for lymphoid malignancies), (iii) diagnosis of the malignancy preceding or following the diagnosis of GD, typically within the same year, with the perquisite that, if the diagnosis of the malignancy followed the diagnosis of the GD, the patient should have not received immunosuppressive therapy for GD. Conversely, if the diagnosis of the malignancy preceded the diagnosis of GD, the patient should not have received chemotherapy or immunotherapy. Exclusion criteria: (i) patients with multiple myeloma and other plasma cell dyscrasia-induced GD, (ii) patients with a history of GD, who were diagnosed with cancer after treatment with immunosuppressants, (iii) patients who received the diagnosis of GD after the diagnosis of malignancy and had received chemotherapy and/or immunotherapy.

Patients’ demographics, biochemistry indexes including serum creatinine and the corresponding estimated glomerular filtration rate (eGFR), 24 h urine protein excretion, findings provided by microscopic urine analysis, hematological and serological tests (antinuclear antibodies (ANA), anti-double-stranded DNA (dsDNA), complement measurements (C3, C4), antineutrophil cytoplasmic autoantibodies (ANCA), anti-glomerular basement membrane autoantibodies, rheumatic factor at the time of kidney biopsy and thereafter every 3 months or by clinical indication were recorded. Histopathology findings were recorded, along with the extra-renal manifestations, therapies given for GDAM, and related responses. The main target of GD management was tumor resection and/or chemotherapy. When GDAM was manifesting as rapidly progressive glomerulonephritis (RPGN), with or without life-threatening symptoms (i.e., alveolar hemorrhage), a short course of immunosuppression was administered, including intravenous cyclophosphamide (500–750 mg/m^2^) with pulses of glucocorticoids, and/or plasma exchange. When used, plasma exchange was initiated for a total of 7 times for 2 weeks, with approximately 1 time the predicted plasma volume (estimated by the following formula: [0.065 × body weight (kg)] × [1 − hematocrit]) per session, using freshly frozen plasma combined with human albumin as the replacement solution. Likewise, for patients with severe nephrotic syndrome resistant to diuretics, who might receive cyclosporine, in a dose of 3 mg/kg of body weight, C_0_ and C_2_ levels were determined every three months.

Outcomes of interest included remission, end-stage kidney disease (ESKD), and death. Remission of GDAM was defined (i) for patients with nephritic syndrome and/or RPGN, as the improvement or stabilization of renal function combined with the resolution of glomerular hematuria and the cessation of hemodialysis and (ii) for patients with nephrotic syndrome, as the decrease in 24 h proteinuria, sustained and >50% of the initial measurement, which remained below the nephrotic range, combined with the disappearance of signs or symptoms of edema. The need of chronic dialysis was defined as ESKD. Estimation of GFR was done using the Modification of Diet in Renal Disease formula [6].

### 2.2. Renal Histopathology Methods

Formalin-fixed and paraffin-embedded tissue sections were prepared for evaluation. Thirteen sections per paraffin block were obtained for light microscopy examination. Three eosin/hematoxylin-stained slides, as well as Periodic Acid Schiff (PAS), Silver, Masson and Congo-Red histochemical stains were evaluated in each case. Immunofluorescence examination on frozen tissue for the detection of immunoglobulins, such as IgG, IgA, and IgM, complement components C3 and C1q, and k and l light chains (DAKO FITC, Polyclonal Rabbit 1/50 dilution), was performed in each case. Slides were examined under a NIKON ECLIPSE 80i Immunofluorescence Microscope with a digital camera. In addition, a small part of the renal cortex was kept in glutaraldehyde solution 2.5%. In case there was not a clear-cut diagnosis from the other two aforementioned methods or clinical indications required electron microscopy studies, this specimen was proceeded adequately for electron microscopy examination with a FEI Morgagni 268 Electron Microscope equipped with a digital camera.

## 3. Results

### 3.1. Description of Patient Population

A total of 41 cases with biopsy-proven GDAM were studied, including 28 (68.3%) patients with solid tumors and 13 (31.7%) with lymphoid malignancies. All included patients were Caucasians, with a mean age of 63.3 (±10.7) years at the time of the diagnostic kidney biopsy. There were 25 (60.9%) males. Patients were initially admitted in the internal medicine department or the hematology section, and during the workup, nephrology consultation was requested, due to the discovery of abnormal renal indexes.

### 3.2. Characteristics Related to GD

Forty-one consecutive patients were identified as having GDAM, accounting for 3.2% of the native kidney biopsies performed in the period 2008–2018. Twenty-four cases (58.5%) presented with new onset of nephrotic syndrome, while eight (33.3%) of them showed also some degree of glomerular hematuria. Sixteen (39.1%) additional patients had newly diagnosed nephritic syndrome, of whom six (37.5%) presented acute glomerulonephritis, six (37.5%) RPGN (Table 1 and Table 2), and the remaining variable degrees of glomerular hematuria and proteinuria. The mean 24 h protein excretion was 5.200 (±1.900) mg in patients with nephrotic syndrome and 907.5 (±519.7) mg in patients with nephritic syndrome, while among patients with acute nephritic syndrome, there were two with nephrotic-range proteinuria. Overall, 20 (48.8%) patients presented with some degree of renal dysfunction or experienced acute renal failure within a few weeks after the diagnosis of GDAM. Mean serum creatinine and albumin, for the total cohort, at the time of the diagnostic biopsy, were 2.3 mg/dL (range 0.6–9.6 mg/dL) and 2.9 (±0.7) mg/dL, respectively. Six patients became dialysis-dependent soon after the diagnosis, five with acute nephritic syndrome and one with nephrotic syndrome. One-third of the patients had positive serological findings, including 7/41 (17%) patients who tested positive for ANA antibodies and 5/41 (12.2%) patients who tested positive for ANCA antibodies. Notably, all cases with ANCA antibodies were against myeloperoxidase (MPO). With respect to the clinical picture, 13 (31.7%) patients had extra-renal manifestations, i.e., pulmonary hemorrhage, hemoptysis, purpura, arthralgias, mononeuritis multiplex, and hemorrhagic colitis. Nineteen (46.3%) patients received immunosuppressive therapy for GDAM. Of these, 13 (68.4%) had acute nephritic syndrome with significant renal dysfunction and/or RPGN and dialysis dependence, while the rest had severe nephrotic syndrome, not remitted despite tumor removal or chemotherapy (Table 1 and Table 2). Notably, all patients with ANCA antibodies presented with acute glomerulonephritis and/or RPGN.

### 3.3. Histopathology Findings

**Figure 1:**
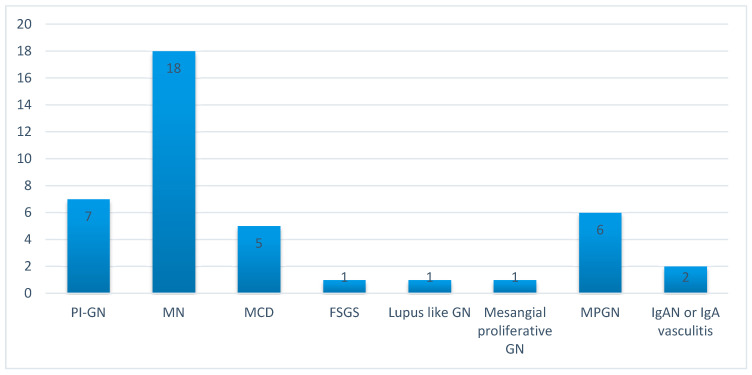
Distribution of histopathological diagnoses revealed by the kidney biopsy in the population of patients with GDAM.

Histopathological evaluation of the kidney biopsy specimens revealed MN in 18 cases (43.9%), membranoproliferative glomerulonephritis (MPGN) in 6 (14.6%), PI-GN in 7 (17.0%), minimal change disease (MCD) in 5 (12.2%), IgA nephropathy with crescent formation or IgA angiitis in 2 (4.9%), lupus-like (WHO class III) glomerulonephritis in 1 (2.4%), mesangial proliferative glomerulonephritis in 1 (2.4%), and focal segmental glomerulosclerosis (FSGS) in 1 (2.4%) (Figure 1).

### 3.4. Characteristics Related to the Malignancy

**Figure 2:**
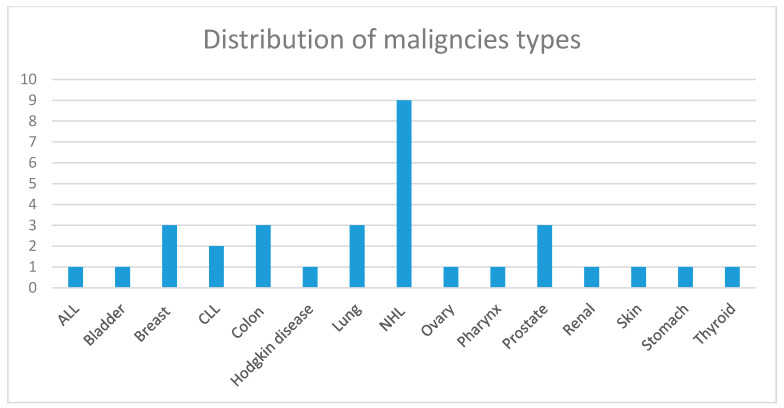
Distribution of the types of malignancy.

The diagnosis of GDAM was established concomitantly with the diagnosis of the malignancy in 14 (34.1%) cases, while in 13 (31.7%), the diagnosis of GDAM preceded the diagnosis of the malignancy, and in 14 (34.1%), it followed the diagnosis of cancer. Among patients with solid tumors (Table 2), the most frequent primary sites of cancer were colon (17.85%), lung (17.85%), breast (14.3%), and prostate (10.7%) (Figure 2), while the most frequent histological type was adenocarcinoma. Patients with lymphoid malignancies (Table 1) included nine cases with non-Hodgkin lymphomas (69.2%), one with Hodgkin disease, and three (23.1%) with leukemia (one acute and two chronic lymphocytic leukemia) (Figure 2). At the time of GDAM diagnosis, eight (19.5%) patients had evident metastatic disease, two in the brain, four in lymph nodes, one in the bladder, and one in the skin. Twenty-one patients (51.2%) underwent surgery, and 30 patients (73.1%) received chemotherapy and/or hormone therapy in addition to surgery or alone. One patient decided not to be treated for the malignancy.

### 3.5. Patient and Renal Survival

Patient survival: During a mean follow-up time of 36.1 (±28.3) months, eight patients (19.5%) died. For four of them, the cause of death was related to metastatic disease, for three to complications linked to therapy, and for one to a cardiovascular event. Two patients were lost in the follow-up. In this series of patient, survival did not differ if GD was documented at the same time as the malignancy, prior, or after it.

**Figure 3:**
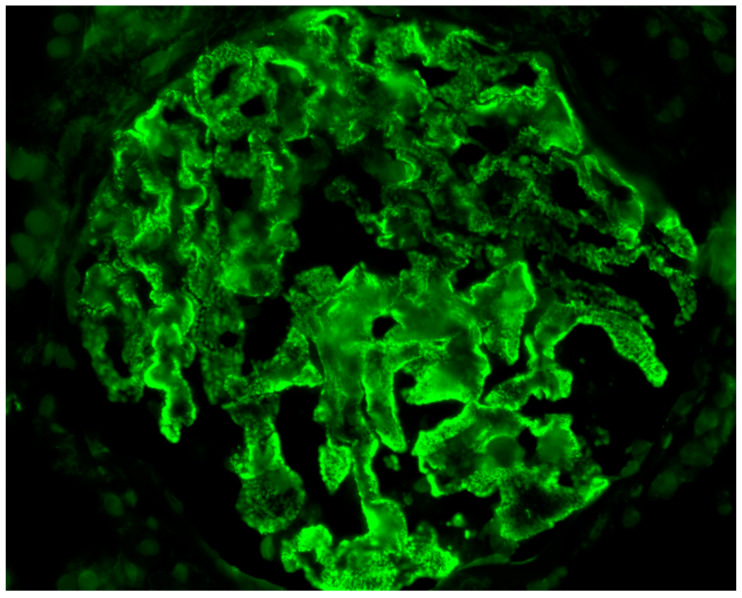
Immunofluorescence image of a kidney biopsy (patient #7, Table 2) showing IgG staining along the glomerular capillary wall, diagnostic of a membranous nephropathy (IgG × 400).

**Figure 4:**
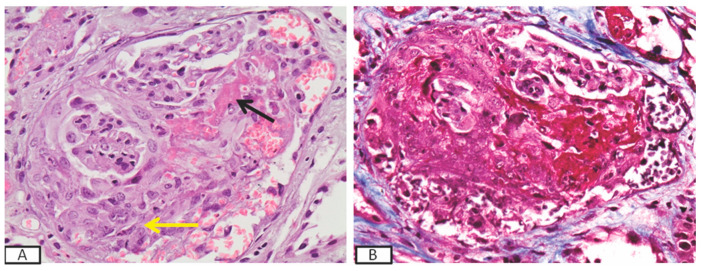
Kidney biopsy of a patient (p. #2, Table 2) who presented with rapidly progressive glomerulonephritis and developed pulmonary–renal syndrome, showing a large cellular crescent (yellow arrow) and segmental fibrinoid necrosis (black arrow) in the glomeruli (**A**) (H&E × 400) by light microscopy and bright red staining of the fibrinoid necrosis in Masson trichrome stain, (Masson × 400) (**B**).

Outcome of GD: Nine (21.95%) patients ended up in chronic dialysis. Five of them had initially presented with severe nephrotic syndrome, which was associated with non-Hodgkin lymphomas in three occasions, acute lymphoblastic leukemia in one occasion, and ovary cancer in the remaining one. Nephrotic syndrome was caused by MN in three (Figure 3) of them and by MCD in one. All three patients experienced prolonged acute renal insufficiency, as a result of the hemodynamic changes, which caused non-resolved acute tubular necrosis, leading to permanent dialysis. All patients, except one, who denied treatment for his lymphoma, received chemotherapy, with no change in the renal function status, while the patient with ovary cancer had a tumor resection as well. One patient, who had recently received the diagnosis of prostate cancer and presented with ANCA-associated RPGN and pulmonary–renal syndrome (Figure 4), remained dialysis-dependent until death, despite immunosuppressive treatment. Three additional patients with nephritic syndrome, caused by MPGN, lupus-like glomerulonephritis, and PI-GN, responded to immunosuppressive therapy and achieved remission but later they ended up in ESKD due to extended, chronic renal injury. Overall, 24 (58.5%) patients achieved remission, in a mean time of 15.7 (±11.25) months. At the end of the observation period, the eGFR of the survivors, who had achieved remission of the GD, was 1.28 (±0.75) mg/dL.

## 4. Discussion

Among patients with GDAM, in our institution, MN was the most prevalent histopathological diagnosis (43.9%). The proportion of patients with GDAM who had MN accounted for the 14.9% of the total cohort of patients with MN diagnosed during the same period. Most of the patients with nephrotic syndrome due to MN had also a solid tumor. The association of MN with solid tumors is well known [7,8,9,10]. A meta-analysis, which included 785 patients, found that the estimated prevalence of cancer among patients with MN was 10% [11], with lung cancer being the most common type of malignancy in those patients. Lefaucher et al. reported 240 patients with MN, 24 of whom received a diagnosis of cancer at the time of the diagnostic kidney biopsy or within the first year [4]. In the same study, it was shown that the incidence of cancer in those patients was 10 times higher than in the general population. In agreement with the study by Leeaphon et al., the proportion of MN associated with lymphoid malignancies was not insignificant in our series of patients [11]. Most of our patients achieved remission of the nephrotic syndrome following tumor resection and/or chemotherapy, pointing to a clear correlation between remission of cancer and remission of the nephrotic syndrome, as previously reported [4]. Pathogenetic pathways which have been suggested [3] to underlie the connection of MN and cancer include (i) the formation of autoantibodies against a tumor antigen with analogous immunological properties to those of an antigen residing within the podocyte, which results in in situ immune complex production; (ii) the production of circulating immune complexes by circulating tumor antigens; (iii) the reaction of circulating antibodies with tumor antigens which are stuck in the glomerular membrane [3]. If a detailed cancer screening in patients with newly diagnosed MN is warranted prior to the induction of immunosuppressive therapy remains in question. The cost–benefit issue has been largely facilitated by the discovery of the phospholipase A_2_ receptor (PLA_2_R) antibody [12], which is strongly associated with idiopathic MN. Among our patients with MN associated with malignancies, 93.3% of the tested patients were negative for the PLA_2_R antibody. Thus, employment of this test, combined with an overall clinical assessment, is proven very helpful in providing a first filter to identify patients who need a more thorough screening for malignancies.

Furthermore, as seen in our study, 12 out of 41 patients had positive serological findings, among which, 5 with lymphoid malignancies and membranoproliferative glomerulonephritis presented ANA, whereas, considering the patients with solid tumors, 5 presented PI-GN, histopathologically associated with MPO-ANCA, 1 PI-GN and ANA, and finally 1 lupus-like histopathologic findings and ANA. PI-GN was exclusively associated with solid tumors, while MPGN was mostly found in patients with lymphoid malignancies. The association of PI-GN with solid tumors has been repeatedly reported [13,14,15,16,17]. Biava et al. reported seven cases of RPGN associated with coexisting malignancies [13]. Several reports, however, highlight the fact that due to the severity of the disease, which may follow a life-threatening course [18], cancer is discovered after the diagnosis of vasculitis, although it runs concurrently [2]. Among six patients with PI-GN in our study, only one was ANCA-negative, while all the remaining patients were MPO-ANCA-positive. Observations in patients with ANCA vasculitides, who had exposure to specific environmental factor, such as drugs or thyroid disease, showed that Perinuclear (P)/MPO-ANCA specificity was more frequent than cytoplasmic (C)/ proteinase (PR3)-ANCA [19,20]. Similarly, among several reports of patients with PI-GN and paraneoplastic syndromes [17,21,22,23,24,25,26,27,28,29], all but one were found to have P/MPO-ANCA in their circulation. Mechanisms probably implicated in cases with vasculitis associated with tumors include the effect of tumor-associated antigens, antibodies, and products reacting with the capillary walls, inducing inflammation, as well as the direct effects of tumor cells on the endothelium, the potential of polyclonal activation of B lymphocytes and induction of monoclonal immunoglobulin activity, and the formation of antibodies directed toward endothelial antigens [30]. In this regard, clinical observations and experimental evidence steadily indicate that ANCA are pathogenic. The pathogenesis of ANCA vasculitis is considered multifactorial, with renal histopathology of patients with PI-GN showing activated neutrophils present in affected glomeruli, and the number of activated intraglomerular neutrophils correlating with the severity of renal injury and the level of renal dysfunction [31]. In vitro, ANCA can activate cytokine-primed neutrophils, causing an oxidative burst, degranulation, release of inflammatory cytokines, and damage to endothelial cells. Thus, acute vascular inflammation may be induced when resting neutrophils that have ANCA autoantigens sequestered in cytoplasmic granules are exposed to priming factors such as cytokines induced by infection or factors released by complement activation. This, in turn, causes the release of ANCA antigens on the surface of neutrophils and in the microenvironment around them [32]. The optimal therapy for RPGN in the setting of cancer is not known. Immunosuppressive agents have the theoretical attendant risk of accelerating the development of malignancy and its spreading. However, arguments justifying their employment include: (i) rescue therapy for life-threatening-conditions, (ii) restriction of the standard scheme by using low-dose intravenous cyclophosphamide plus glucocorticoids, typically for three months, (iii) use of cyclophosphamide by oncologists for the treatment of certain malignancies [33,34,35]. Thus, oncology consultation combined with individualized approach and management are mandatory for such cases.

Limitations of this study include the small number of patients, which is related to the fact that these disorders are particularly rare and their diagnosis can be very difficult, due to delayed onset of symptoms for certain malignancies and the presence of other secondary causes of kidney disease. We included only patients who received both diagnoses either concomitantly or within the same year, and thus, occasional patients, who manifested the GDAM out of this period, may have been missed. The probability of random incidence of two independent events cannot be excluded, but the closer the diagnosis between the two, the lower the probability of concurrency by chance. Yet, the lack of expected remission of GD after tumor resection or treatment was probably related to the fact that malignancies represent systemic diseases with obvious and occasionally not evident metastases.

In conclusion, in this series of patients with GDAM, serological findings such as ANA positivity and detection of ANCA antibodies were relatively frequent, implying a potential connection with immunopathogenesis, especially in the case of PI-GN. In contrast, the absence of anti-PLA_2_R antibodies in the vast majority of patients with MN indicates the secondary cause of nephrotic syndrome, i.e., the malignancy, and thus may be very helpful in terms of clinical management to identify those patients with MN who need a more extensive workup for malignancies. Prompt diagnosis and individualized management, in accordance with the specific characteristics of the patient, the malignancy, and GD histopathology, are critical for these patients.

## 5. Statement of Ethics

This is a retrospective, descriptive study, which was performed in compliance with the declaration of Helsinki for published research. No other ethics committee approval was required for this study. This article does not contain any studies with human participants or animals performed by any of the authors

## Figures and Tables

**Table 1:** Characteristics of the patients with glomerular diseases associated with malignancies (GDAM) presenting lymphoid malignancies at the diagnostic kidney biopsy.

Patient	Age (Years)	Renal Histopathology	Type of Malignancy	Clinical Presentation of the Glomerular Disease	Ser. Creat. (mg/dL)	24 h Protein Excretion (mg)	Positive Serological Findings
P. 1	61	MPGN	NHL (Malt)	Acute GN	1.9	9905	ANA
P. 2	76	MPGN	NHL (B cell)	Rapidly progressive GN	4.7	6641	ANA
P. 3	46	MPGN	NHL (T cell)	Nephritic syndrome	1.1	2000	-
P. 4	52	MN	NHL (B cell)	Nephrotic syndrome	1.2	3200	-
P. 5	43	MPGN	NHL (Malt)	Nephritic syndrome	0.98	1100	ANA
P. 6	62	IgAN	CLL	Acute GN	1.4	1020	-
P. 7	57	MPGN	NHL (B cell)	Nephritic syndrome	0.6	850	ANA
P. 8	59	MN	ALL	Nephrotic syndrome-AKI	7.7	12,000	-
P. 9	79	MPGN	NHL (T cell)	Acute GN	1.4	299	ANA
P. 10	79	MN	NHL (B cell)	Nephrotic syndrome	0.9	7500	-
P. 11	66	MCD	NHL (B cell)	Nephrotic syndrome	0.9	4200	-
P. 12	66	MN	CLL	Nephrotic syndrome-AKI	2.8	9540	-
P. 13	56	MCD	Hodgkin disease	Nephrotic syndrome-AKI	1.5	16,900	-

Abbreviations: Ser. Creat.: Serum creatinine, MPGN: Membranoproliferative glomerulonephritis, MN: Membranous nephropathy, IgAN: IgA Nephropathy, MCD: Minimal change disease, PI-GN: pauci-immune glomerulonephritis, GN: glomerulonephritis, NHL: Non-Hodgkin lymphoma, ALL: Acute lymphoblastic leukemia, CLL: Chronic lymphoblastic leukemia, ANA: Antinuclear autoantibodies, MPO-ANCA: Antineutrophil-cytoplasmic autoantibodies against myeloperoxidase, AKI: Acute kidney injury.

**Table 2:** Characteristics of the patients with GD associated with solid tumors.

Patient	Age (Years)	Renal Histopathology	Type of Malignancy	Clinical Presentation of the Glomerular Disease	Ser. Creatinine (mg/dL)	24 h Protein Excretion (mg)	Positive Serological Findings
P. 1	59	PI-GN	Breast cancer	Rapidly progressive GN	3.3	1200	-
P. 2	70	PI-GN	Prostate cancer	Pulmonary-renal syndrome	9.6	120	MPO-ANCA
P. 3	59	MCD	Ovary cancer	Nephrotic syndrome	0.8	6670	-
P. 4	59	MN	Colon cancer	Nephrotic syndrome	0.8	4000	-
P. 5	59	MN	Lung cancer	Nephrotic syndrome	1.2	4900	-
P. 6	71	PI-GN	Pharynx cancer	Rapidly progressive GN	6.2	1435	MPO-ANCA
P. 7	54	MN	Lung cancer	Nephrotic syndrome	0.7	10,300	-
P. 8	44	MN	Thyroid cancer	Nephrotic syndrome	0.8	3700	-
P. 9	82	MCD	Prostate cancer	Nephrotic syndrome-AKI	2.8	6900	-
P. 10	65	MN	Colon cancer	Nephrotic syndrome	0.6	3200	-
P. 11	80	MN	Colon cancer	Nephrotic syndrome	0.9	7600	-
P.12	37	Lupus-like GN	Kidney cancer	Nephritic syndrome	0.7	4300	ANA
P. 13	67	MN	Breast cancer	Subnephrotic proteinuria	0.9	2200	-
P.14	49	Mesangial proliferative GN	Breast cancer	Glomerular hematuria, proteinuria	0.8	625	-
P.15	67	PI-GN	Bladder cancer	Acute GN	3.3	810	MPO-ANCA
P. 16	67	PI-GN	Prostate cancer	Acute GN	2.7	1200	MPO-ANCA
P. 17	68	PI-GN	Skin cancer	Acute GN	2.6	1030	MPO-ANCA
P. 18	70	FSGS-tip lesion	Colon cancer	Nephrotic syndrome	1.0	8300	-
P. 19	82	PI-GN	Stomach (neuroendocrine)	Rapidly progressive GN	8.5	5400	-
P. 20	73	MN	Lung cancer	Nephrotic syndrome-AKI	7.5	8341	-
P.21	64	IgA vasculitis	Lung cancer	RPGN	8.1	2831	-
P.22	50	MN	Thyroid cancer	Nephrotic syndrome	0.76	7700	-
P.23	67	MN	Lung cancer	Nephrotic syndrome	0.79	10,500	-
P.24	68	MN	Kidney cancer	Nephrotic syndrome	2.05	8100	-
P.25	73	MN	Breast cancer	Nephrotic syndrome	0.66	4500	-
P.26	59	MN	Mesothelioma	Nephrotic syndrome	0.6	10,314	-
P.27	60	MN	Colon (neuroendocrine)	Nephrotic syndrome	0.6	6350	-
P.28	72	MCD	Ovary cancer	Nephrotic syndrome	0.6	15,100	ANA

Abbreviations: MN; Membranous nephropathy, MCD; Minimal change disease, FSGS; Focal Segmental Glomerulosclerosis, PI-GN; pauci-immune glomerulonephritis, GN; glomerulonephritis, ANA; Antinuclear autoantibodies, MPO-ANCA; Antineutrophil-cytoplasmic autoantibodies against myeloperoxidase, AKI; Acute kidney injury IgAN; IgA Nephropathy.

## Abbreviations

GD	glomerular disease
GDAM	glomerular disease associated with malignancy
PLA_2_R	phospholipase A_2_ receptor
ANCA	antineutrophil cytoplasmic autoantibodies
MPO	myeloperoxidase
PR3	proteinase 3
ESKD	end stage kidney disease
ANA	anti-nuclear autoantibodies
PI-GN	pauci-immune glomerulonephritis
RPGN	rapidly progressive glomerulonephritis
GFR	glomerular filtration rate
MPGN	membranoproliferative glomerulonephritis
MN	membranous nephropathy
IgAN	IgA nephropathy
MCD	minimal change disease
GN	glomerulonephritis
FSGS	focal segmental glomerulosclerosis
